# Analysis of the first ten years of FDA’s rare pediatric disease priority review voucher program: designations, diseases, and drug development

**DOI:** 10.1186/s13023-024-03097-x

**Published:** 2024-02-25

**Authors:** Catherine Mease, Kathleen L. Miller, Lewis J. Fermaglich, Jeanine Best, Gumei Liu, Erika Torjusen

**Affiliations:** 1 Office of Orphan Products Development, Office of the Commissioner, US Food and Drug Administration, 10903 New Hampshire Ave, Silver Spring, MD 20993 USA; 2 Office of Pediatric Therapeutics, Office of the Commissioner, US Food and Drug Administration, 10903 New Hampshire Ave, Silver Spring, MD 20993 USA; 3 Office of Therapeutic Products, Center for Biologics Evaluation and Research, US Food and Drug Administration, 10903 New Hampshire Ave, Silver Spring, MD 20993 USA

**Keywords:** Rare pediatric diseases, Food and Drug Administration, Children, Designations, Drug development

## Abstract

**Background:**

The Rare Pediatric Disease (RPD) Priority Review Voucher (PRV) Program was enacted in 2012 to support the development of new products for children. Prior to requesting a voucher, applicants can request RPD designation, which confirms their product treats or prevents a rare disease in which the serious manifestations primarily affect children. This study describes the trends and characteristics of these designations. Details of RPD designations are not publicly disclosable; this research represents the first analysis of the RPD designation component of the program.

**Results:**

We used an internal US Food and Drug Administration database to analyze all RPD designations between 2013 and 2022. Multiple characteristics were analyzed, including the diseases targeted by RPD designation, whether the product targeted a neonatal disease, product type (drug/biologic), and the level of evidence (preclinical/clinical) to support designation. There were 569 RPD designations during the study period. The top therapeutic areas were neurology (26%, n = 149), metabolism (23%, n = 131), oncology (18%, n = 105). The top diseases targeted by RPD designation were Duchenne muscular dystrophy, neuroblastoma, and sickle cell disease. Neonatology products represented 6% (n = 33), over half were for drug products and 38% were supported by clinical data.

**Conclusions:**

The RPD PRV program was created to encourage development of new products for children. The results of this study establish that a wide range of diseases have seen development—from rare pediatric cancers to rare genetic disorders. Continued support of product development for children with rare diseases is needed to find treatments for all children with unmet needs.

## Introduction

Product development for pediatric populations faces numerous challenges. These include difficulties in patient enrollment and retention, dosing and safety challenges, and ethical considerations [1–4]. Additionally, the market for therapeutics in pediatric populations is smaller than for the adult population and therefore may be less attractive to for-profit developers [5]. As a result, there is a significant dearth in the number of approved drugs and biologics for pediatric use [6, 7]. While financial incentive programs exist, such as those created by the Orphan Drug Act, these challenges are especially acute in rare pediatric diseases [8]. One way to address this unmet need is through the provision of additional financial incentives for companies to pursue pediatric product development.

Over the decades, U.S. Congress has passed numerous laws to encourage or require the development of therapeutics for children [9]. These include the Best Pharmaceuticals for Children Act (BPCA) of 2002 (which provides incentives to drug developers who voluntarily complete pediatric studies for their product) and the Pediatric Research Equity Act (PREA) of 2003 (which allows the US Food and Drug Administration [FDA] to require pediatric studies for certain products) [10–12]. However, products for rare diseases with orphan drug designation, exclusive of pediatric cancers, are exempt from the requirements of PREA [13, 14]. Therefore, an additional incentive program specific to rare diseases in children, the Rare Pediatric Disease (RPD) Priority Review Voucher (PRV) Program, was established in 2012 with enactment of the Food and Drug Administration Safety and Innovation Act [15–17].

PRVs are financial incentives to drug and biologic development, which are also awarded for approval of certain marketing applications for tropical diseases and medical countermeasures [16, 18, 19]. For RPD, companies may be awarded an RPD PRV when their rare pediatric disease product application is approved by the FDA [16]. The PRV can be redeemed for a priority review (a goal of six months of regulatory review time) instead of a standard review (a goal of ten months of regulatory review time) for a subsequent product application submitted for FDA approval or can be transferred to a third party [20]. The reported purchase prices of PRVs to third parties averages about $100 million (range $67.5 million to $350 million) [21, 22].

Prior to companies receiving an RPD PRV, a determination must be made that a drug or biologic is for a rare pediatric disease. Although RPD designation is not required prior to requesting an RPD PRV, the FDA strongly encourages companies to submit a request for RPD designation prior to submission of a potential rare pediatric disease product application. All applicants must document in their voucher request how their application meets RPD PRV eligibility criteria, including support that their drug or biologic is for the prevention or treatment of a rare pediatric disease, which may be established by RPD designation [16, 23].

To be granted RPD designation, an applicant must submit a request that includes data supporting the proposed mechanism of action of the drug or biologic (e.g., clinical, preclinical: in vivo, and preclinical: in vitro). Additionally, the applicant must demonstrate that the disease is a “rare pediatric disease,” defined as a life-threatening disease in which the serious or life-threatening manifestations primarily affect individuals aged from birth to 18 years, and the total prevalence of the disease, including adults and children, affects fewer than 200,000 people [16, 23]. The RPD designation portion of the RPD PRV program is administered by the Office of Orphan Products Development in collaboration with the Office of Pediatric Therapeutics within the Office of the Commissioner at the FDA.

The primary objective of this study was to provide a 10-year retrospective analysis of RPD designations from the program initiation in 2013. This is the first time this data is being made available as RPD designations are not publicly disclosable. An analysis of the first decade of RPD designations provides important information for stakeholders, as limited information is available in the public domain and the RPD PRV program is due to sunset in 2024 if Congressional action is not taken to renew it. This retrospective analysis additionally builds on previous research that investigates the impact of legislation on rare pediatric product development [15, 24].

## Methods

We used an internal FDA database to analyze all RPD designations, from 2013 to 2022. The dataset included: (1) date of designation; (2) product name; (3) disease description; (4) approval; and (5) voucher status.

The dataset also included whether the product was therapeutic, preventative, or diagnostic for management. Per FDA’s RPD PRV guidance: “[a]n application may qualify as a rare pediatric disease product application if it is for a drug or biologic that is a diagnostic for the management of a disease or condition. We note, however, that such diagnostic products must be the subject of a NDA [new drug application] or BLA [biologic license application] to qualify as a rare pediatric disease product application, as diagnostic products that are the subject of medical device applications are not eligible for a rare pediatric disease[s] priority review voucher. An application for a drug for the initial diagnosis of a disease or condition will not qualify as a rare pediatric disease product application” [16].

Finally, the dataset included whether the designation was subject to the 60-day statutory review deadline or not. A designation request receives a 60-day FDA review clock when it is submitted concurrently with a request for fast-track designation or orphan drug designation [8, 25]. Those requests submitted without either additional designation request do not have a statutory review goal date, but FDA aims to respond to such requests in a timely manner.

Using this initial dataset, we constructed multiple additional variables for analysis. We assigned a therapeutic area to each designation, which was based primarily upon the general disease process (e.g., oncology, infectious disease) and secondarily, the most affected organ system [26].

All RPD disease descriptions were converted from longer phrases (e.g., “For the treatment of Angelman syndrome”) to simplified disease terms (e.g., Angelman syndrome) to allow for aggregation across designations. To confirm that each disease term was a recognized and described disease or condition, we used a uniform system of disease terminology (“Mondo”) created to facilitate integration and consistency of disease nomenclature across various ontology resources [27].

Additionally, we created a dichotomous variable to identify designations for neonatology conditions. Neonatology conditions were defined as disorders in children up to 44 weeks post-menstrual age that were: (1) conditions related to prematurity and physiologic immaturity, or (2) conditions in which the serious or life-threatening manifestations primarily affect neonates or typically present during the neonatal period and the time to intervene occurs during the neonatal period [28]. Metabolic diseases that require lifelong treatment were not included in the neonatology definition.

For drug and biologic descriptions, a product type category was constructed by determining whether the designated products were biologic or drug products. Within the biologic product type category, we further identified all vaccines, monoclonal antibodies, cell therapies, and gene therapies. We also identified all antisense oligonucleotide products within the drug product type category.

We constructed a variable to investigate the level of scientific evidence used to support the request for RPD designation. Designations were categorized into those that utilized clinical data, preclinical in vivo data, or preclinical in vitro data, all of which are acceptable to support designation. Clinical evidence was further classified into applicant-derived clinical data, clinical trial data cited from the literature (“cited clinical trial”), or a case study cited from the literature (“cited case study”).

Lastly, we gathered data on annual counts and therapeutic areas of the RPD PRVs that have been awarded based on the approval of a product for a rare pediatric disease.

## Results

**Fig. 1:**
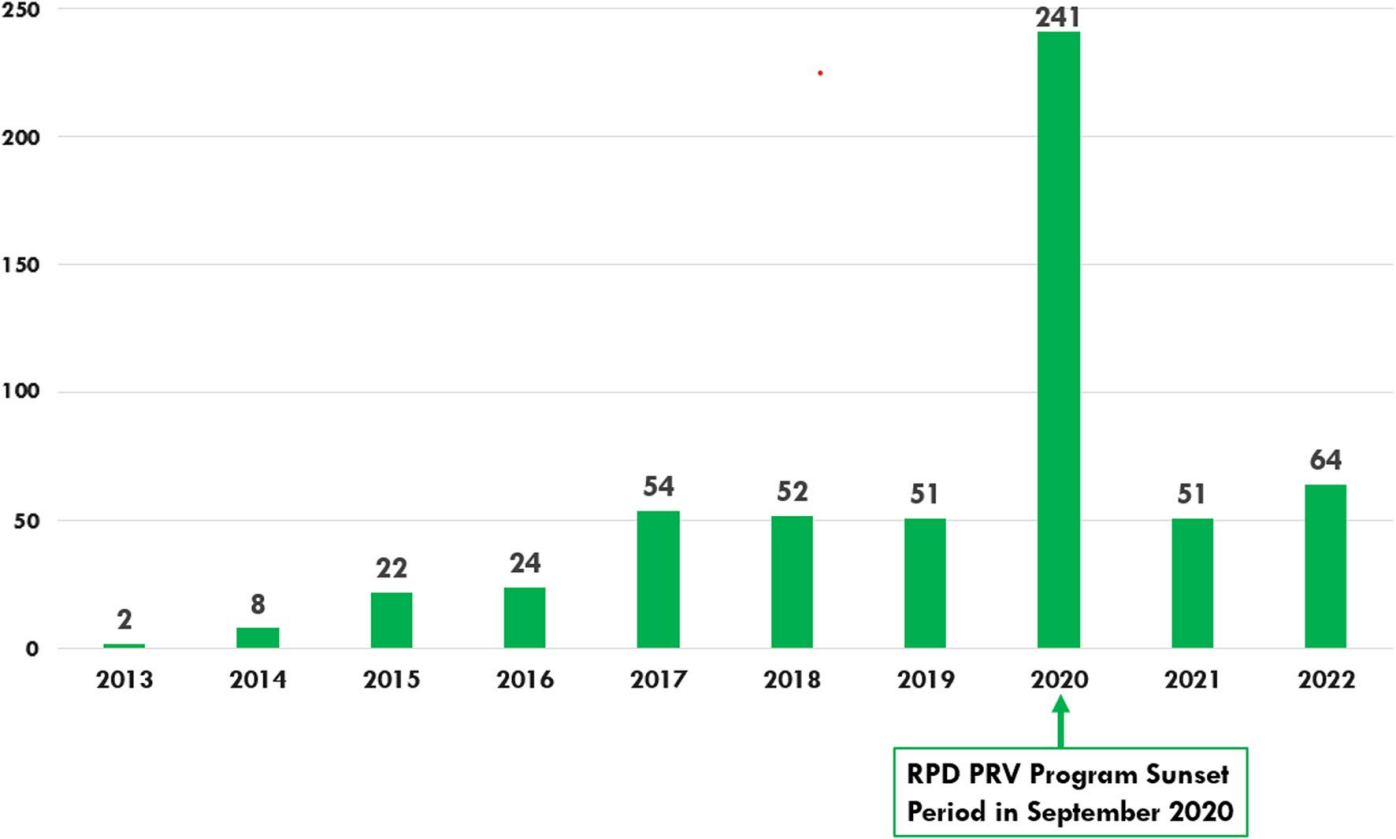
Number of RPD designations per year, 2013–2022 (n = 569). On December 27, 2020, the Rare Pediatric Disease Priority Review Voucher Program was extended. Under the current statutory sunset provisions, after September 30, 2024, FDA may only award a voucher for an approved rare pediatric disease product application if the sponsor has rare pediatric disease designation for the product, and that designation was granted by September 30, 2024. After September 30, 2026, FDA may not award any rare pediatric disease priority review vouchers

There have been 569 RPD designations since the inception of the RPD PRV program through December 31, 2022 (Fig. 1). Annual designation frequency was relatively constant over this period with the exception of 2020 (the year the program was set to begin sunsetting), during which a nearly five-fold increase was observed. This single year accounted for 42% (n = 241) of the total designations.

Fifty-four percent (n = 305) of the designations had a statutory FDA review clock of 60 days and 46% (n = 264) of the designations did not have a review clock. The number of designation requests with a 60-day FDA review clock has steadily increased over the years suggesting that more RPD designation requests are being submitted concurrently with a request for fast-track or orphan drug designation.

### Treatments, prophylactics, and diagnostics for management

Virtually all RPD designations were for treatments (95%, n = 538). Diagnostics for management of diseases comprised 3% (n = 19) of designations and prophylactic products 2% of designations (n = 12).

### Therapeutic areas

The top five therapeutic areas were neurology (26%, n = 149), metabolism (23%, n = 131), oncology (18%, n = 105), hematology (6%, n = 32), and immunology (4%, n = 25) (Table 1). Metabolism had the greatest number of diseases represented with 70 unique diseases targeted by RPD-designated products.

**Table 1:** RPD designations by therapeutic areas, 2013–2022

Therapeutic area	Number of RPD designations	Percentage of total RPD designations (%)	Number of diseases associated with at least 1 RPD designation within therapeutic area	Number of RPD designations per disease within therapeutic area
Neurology	149	26	63	2.4
Metabolism	131	23	70	1.9
Oncology	105	18	22	4.8
Hematology	32	6	8	4
Immunology	25	4	16	1.6
Ophthalmology	22	4	16	1.4
Dermatology	21	4	10	2.1
Pulmonary	15	3	6	2.5
Orthopedics	15	3	6	2.5
Endocrinology	12	2	6	2
Gastroenterology	12	2	7	1.7
Cardiology	9	2	5	1.8
Infectious diseases	9	2	6	1.5
Otorhinolaryngology	5	1	2	2.5
Nephrology/urology	4	1	3	1.3
Transplant	2	< 1	2	1
Pharmacology/toxicology/poisoning/chelators	1	< 1	1	1

### Diseases targeted by RPD designations

A total of 245 diseases were represented in the RPD designations granted in the ten-year period between 2013 and 2022. There were 26 diseases with five or more associated RPD designations, which, in total, accounted for 41% of all RPD designations (n = 233) (Table 2). Diseases associated with only one designated product accounted for 62% (n = 152) of total diseases targeted by RPD designation. The remaining 38% (n = 93) of diseases were associated with two to four RPD designations.

**Table 2:** Distribution of RPD designations per disease

Disease breakdown	Number of diseases	Percentage of total diseases (n = 245) (%)	Percentage of total RPD designations (n = 569) (%)
Number of diseases associated with 1 RPD designation	152	62	27
Number of diseases associated with 2 RPD designations	32	13	11
Number of diseases associated with 3 RPD designations	18	7	9
Number of diseases associated with 4 RPD designations	17	7	12
Number of diseases associated with 5 or more RPD designations	26	11	41

Table 3 presents diseases associated with the most RPD designations. Three of the top five diseases targeted by RPD designation were cancers: neuroblastoma, diffuse intrinsic pontine glioma, and osteosarcoma.

**Table 3:** Diseases associated with at least five RPD designated products (n = 26)

Most designated diseases	Number of RPD designations	Percentage of total RPD designations	Therapeutic area
Duchenne muscular dystrophy	30	5	Neurology
Neuroblastoma	21	4	Oncology
Sickle cell disease	18	3	Hematology
Diffuse intrinsic pontine glioma*	16	3	Oncology
Osteosarcoma	15	3	Oncology
Acute lymphoblastic leukemia	10	2	Oncology
Cystic fibrosis	8	1	Pulmonary
Epidermolysis bullosa	8	1	Dermatology
Congenital isolated hyperinsulinism	7	1	Endocrinology
Dravet syndrome	7	1	Neurology
Ewing sarcoma	7	1	Oncology
Friedreich ataxia	7	1	Neurology
GM2 gangliosidosis^#^	7	1	Metabolism
B-thalassemia	6	1	Hematology
Medulloblastoma	6	1	Oncology
Mucopolysaccharidosis type 1	6	1	Metabolism
Propionic acidemia	6	1	Metabolism
Spinal muscular atrophy	6	1	Neurology
Angelman syndrome	5	1	Neurology
Gaucher disease	5	1	Metabolism
Krabbe disease	5	1	Neurology
Lennox-Gastaut syndrome	5	1	Neurology
Mucopolysaccharidosis type 2	5	1	Metabolism
Netherton syndrome	5	1	Dermatology
Rett syndrome	5	1	Neurology
Rhabdomyosarcoma	5	1	Oncology

*Diffuse intrinsic pontine glioma has also been classified as pediatric-type diffuse high-grade gliomas

^#^GM2 gangliosidosis includes both Tay-Sachs and Sandhoff diseases

21 USC 360bb(a)(2) defines a “rare disease or condition” as: “any disease or condition which (A) affects less than 200,000 persons in the United States, or (B) affects more than 200,000 [sic] in the United States and for which there is no reasonable expectation that the cost of developing and making available in the United States a drug for such disease or condition will be recovered from sales in the United States of such drug.”

### Neonatology

Products designated to treat neonatal conditions represented 6% (n = 33) of the RPD designations. Four neonatal conditions (neonatal seizures, bronchopulmonary dysplasia, necrotizing enterocolitis, and retinopathy of prematurity) are each associated with four RPD designations. These four neonatal conditions comprise nearly half of the neonatology products designated.

### Product type

**Fig. 2:**
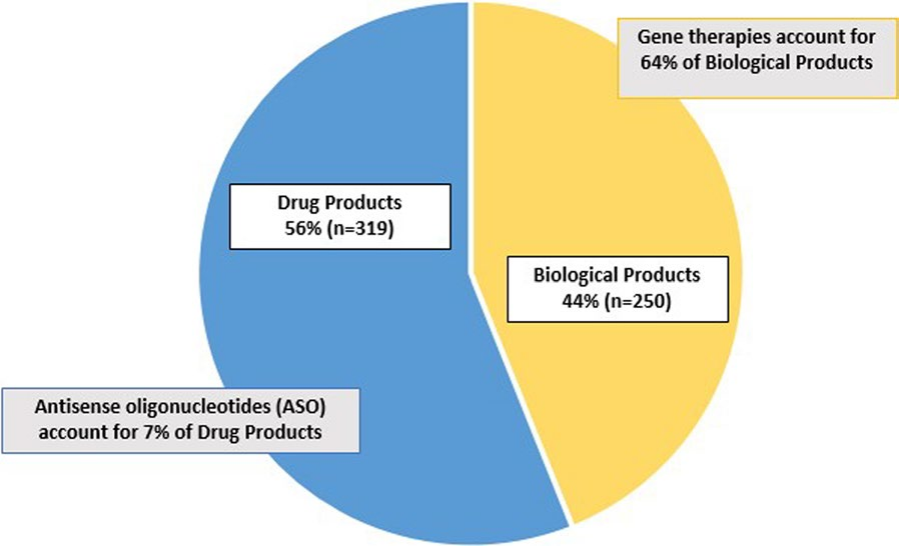
Product type of RPD designations, 2013–2022 (n = 569)

Figure 2 represents the breakdown of drugs versus biologics among the RPD designations. A majority (56%, n = 319) of the designations were for drugs and 44% (n = 250) were for biologic products. There was no change in the relative proportion of drugs versus biologic products over time. Six percent (n = 22) of drugs were antisense oligonucleotides. Sixty-four percent (n = 161) of biologics were gene therapies.

### Level of evidence

**Fig. 3:**
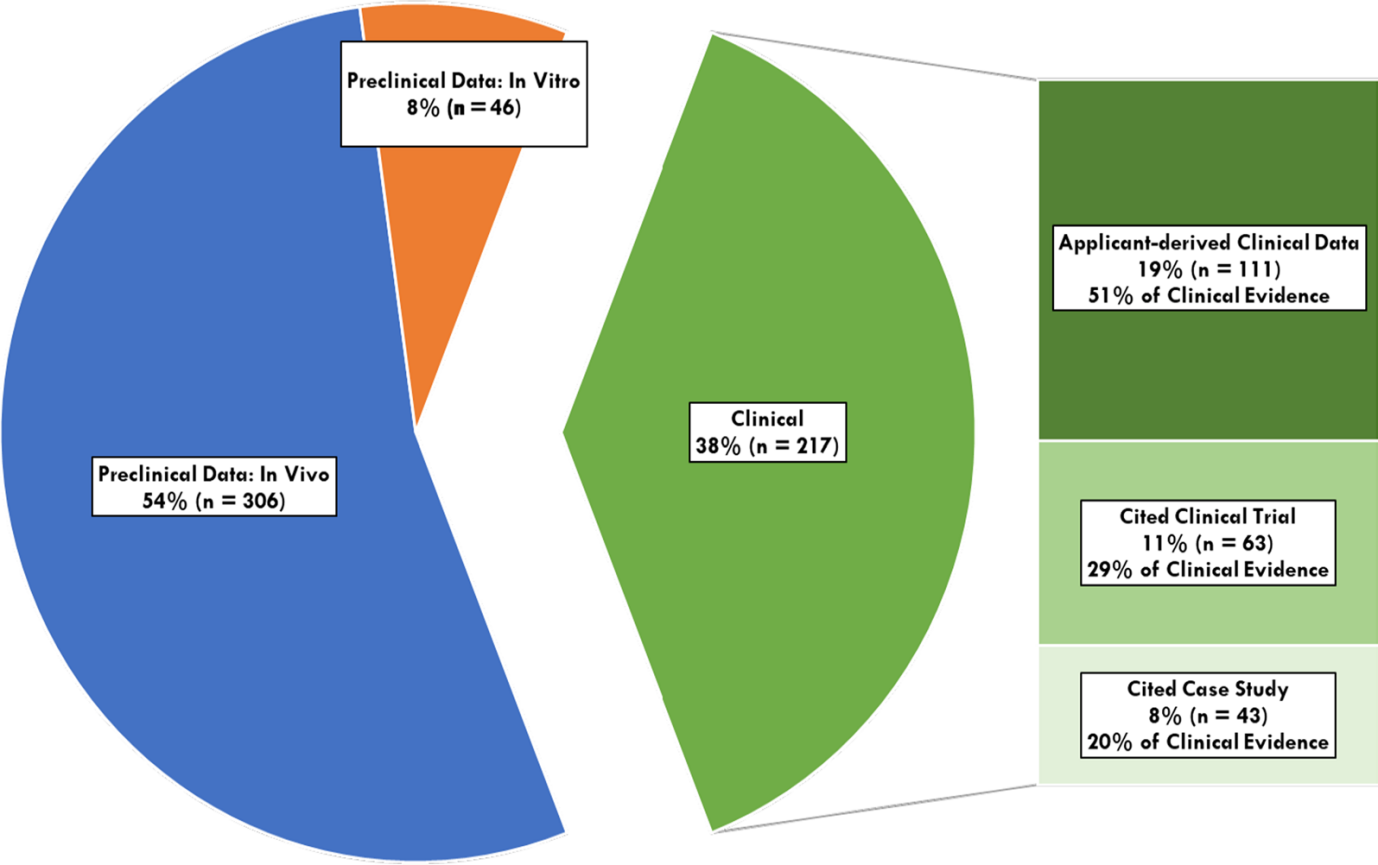
Types of evidence used by applicants to support scientific rationale for RPD designation (n = 569)

The majority of RPD designations, 54% (n = 306), supported their request for designation with preclinical in vivo evidence, followed by 38% (n = 217) providing clinical evidence (Fig. 3). The use of clinical evidence remained relatively stable over time. The majority (51%, n = 111) of clinical evidence originated from applicant-conducted clinical trials. The remaining designations supported by clinical evidence was split between applicants using cited clinical trials (29%, n = 63) and case studies (20%, n = 43).

### Priority review vouchers

There were 38 RPD PRVs awarded from 2014 to 2022 (Table 4). The therapeutic areas with the most awarded PRVs were neurology (n = 13) and metabolism (n = 12), accounting for two thirds of all vouchers. The highest number of RPD PRVs awarded in a single year occurred in 2020 with seven.

**Table 4:** Summary of awarded RPD priority review vouchers, 2014–2022 (n = 38)

	Number of RPD priority review vouchers awarded	Therapeutic area (s)
2014	1	Metabolism (1)
2015	5	Metabolism (4); Oncology (1)
2016	2	Neurology (2)
2017	5	Neurology (2); Metabolism (1); Oncology (1); Ophthalmology (1)
2018	5	Immunology (2); Metabolism (1); Neurology (1); Pulmonary (1)
2019	3	Neurology (2); Pulmonary (1)
2020	7	Metabolism (3); Neurology (3); Oncology (1)
2021	6	Gastroenterology (1); Metabolism (1); Neurology (1); Ophthalmology (1); Orthopedics (1); Pulmonary (1)
2022	4	Neurology (2); Hematology (1); Metabolism (1)

Per Sect. 505 (b)(1) of the FD&C Act or Sect. 351 of the Public Health Service (PHS) Act, the PRV can be redeemed for a priority review instead of a standard review for another drug or biologic submitted for FDA approval after the date of approval of the rare pediatric disease product

## Discussion

Supporting product development for children is a critical public health initiative, given the substantial unmet need. A Congressional Research Service Report explains that: “drug manufacturers are reluctant to test drugs in children because of economic, ethical, legal, and other obstacles. Market forces alone have not provided manufacturers with sufficient incentives to overcome these obstacles.” [29] The RPD PRV program seeks to encourage product development for the pediatric population by providing a financial incentive for companies developing drugs and biologics specifically for children with rare diseases.

In the decade since the RPD PRV Program was introduced, 569 RPD designations were granted for products targeting 245 unique rare pediatric diseases. Almost half (42%) of these designations occurred in one year, 2020. We surmise that this extreme spike occurred because the RPD PRV program was going to begin sunsetting on September 30, 2020. Companies needed to receive their RPD designation before that date to maintain their eligibility for a future PRV. If the program had indeed terminated, no further RPD PRVs would have been granted after September 30, 2022. While this same phenomenon was not seen in 2016 when the RPD PRV program was previously due to sunset, we believe a spike did not occur at that time because the Congressional language in place at the time did not include a date by which designation was required. Spikes in designation activity like that experienced in 2020 may continue in the future if Congressional language includes a date by which designation is required, and if early action is not taken to renew the program resulting in potential uncertainty for sponsors.

Today, the RPD PRV program has not been permanently reauthorized by Congress—it must be renewed periodically by legislative action. This need for continued reauthorization creates unpredictability in long-term planning and resource allocation for companies developing these drugs and biologics, which could potentially lead to less product development in this space.

Our conclusions are fourfold. First, there was a wide range of rare pediatric diseases for which product development has been occurring. There were 245 unique diseases targeted by RPD-designation, and there was no single disease that was the focus of most of the development. Additionally, while four of the top ten diseases most often associated with RPD designated products were cancers, this is congruent with the trends seen in the adult rare cancer space, which have experienced increased development [30].

Second, we find that drugs and biologics intended for use in the neonatal population represent a surprisingly small proportion of all RPD designations (6%). However, it is notable that within this subpopulation, the more frequently encountered diseases are represented in the designations granted. This could indicate the most well studied neonatal diseases are those that see the most translation into product development. The difficulties with developing new drugs and biologics for neonatal patients have been well documented, including: (1) few appropriate animal models; (2) challenging trial designs; and (3) high rates of co-morbidities [31, 32]. To address some of these concerns encountered by neonatal product developers and to encourage product development in this vulnerable population, FDA published a guidance in 2022 to assist product developers who are planning to conduct clinical pharmacology studies in neonatal populations, but more progress must be made in this field [33]. FDA also published a guidance in 2023 to support innovators in approaching neurodevelopment safety studies in neonates [34].

Third, we find that gene therapy products represent more than a quarter (28%) of all designations. As it has been estimated that more than 70% of rare diseases have a genetic etiology and these diseases disproportionately affect children, it is not surprising that this technology type is a frequent RPD designation target [35]. High-profile approvals of gene therapies for pediatric-onset diseases like spinal muscular atrophy and RPE65-related retinal disease could potentially pave the way for gene therapies as a model for both therapeutic and market viability [36, 37].

Finally, we find that a substantial number of designations are supported by clinical data. Nearly 40% of all RPD designations were granted based on clinical evidence. This is important because it suggests that the preliminary results for these products show potential promise and are further along in development than those supported only by preclinical data. Additionally, the proportion of RPD designations supported by clinical evidence is relatively stable over time, indicating that this result is not a legacy from program initiation (i.e., clinical development programs that already existed when the program began).

Studies of the RPD PRV program have found mixed results in discerning whether the program has stimulated development for these diseases [15]. While this study cannot determine any causal relationships, it contributes a more detailed description of the landscape of product development for rare pediatric diseases. The results indicate that while the program has supported the award of 38 PRVs (for products that are approved for the prevention or treatment of a rare pediatric disease) in its first ten years, this represents only 7% of the RPD-designated products that are not yet approved. However, we also acknowledge that the development of new drugs and biologics can take more than a decade and most are ultimately not approved [38]. Therefore, it is expected that drug and biologic approvals would lag behind RPD designations, and approvals being a less common event, would only represent a fraction of the total number of designations.

Future research is needed to assess the overall impact of the RPD PRV program to evaluate other important outcomes (beyond product approvals) such as progression through clinical trials, the impact for developers to secure additional funding (i.e., venture capital and angel funding, grants), and the initiation of natural history studies. For rare pediatric diseases not currently represented in the RPD designation program, additional research is needed to understand the potential barriers to product development to effectively shape future initiatives to address these urgent needs.

### Limitations

While this study analyzes all RPD designations, it may not capture the entire product development pipeline for this population. For example, nonprofits, such as academic researchers, may have little awareness or incentive to apply for this designation, and therefore their research will not be represented in the results. This may limit the generalizability of our study.

Second, while we attempted to make the disease categorization as consistent as possible by using a respective disease ontology and reviewing categorization decisions with all of the study authors, there is inherent subjectiveness in classifying unique diseases.

## Conclusion

The RPD PRV program was created to stimulate the development of new therapies for this historically neglected patient population–children. Prior to receiving a PRV, companies can elect to first receive RPD designation for their product. This research publishes, for the first time, a retrospective assessment of the RPD designation portion of the RPD PRV program. While a wide variety of rare pediatric diseases are represented, most designations are focused in the neurologic, metabolic, and oncologic therapeutic areas. More than a quarter of RPD designations are for gene therapies, and over a third of RPD designations are supported by clinical data. Additional research is needed to evaluate the full impact of the RPD PRV program that extends beyond product approvals. Augmented support for rare pediatric disease product development is needed to address the healthcare inequity facing one of our most vulnerable populations.

## Data Availability

The datasets generated and/or analyzed during the current study are not listed on any public FDA databases.
