# Correction to: Analysis of the first ten years of FDA’s rare pediatric disease priority review voucher program: designations, diseases, and drug development

**DOI:** 10.1186/s13023-024-03136-7

**Published:** 2024-03-21

**Authors:** Catherine Mease, Kathleen L. Miller, Lewis J. Fermaglich, Jeanine Best, Gumei Liu, Erika Torjusen

**Affiliations:** 1 Office of Orphan Products Development, Office of the Commissioner, US Food and Drug Administration, 10903 New Hampshire Ave, 20993 Silver Spring, MD USA; 2 Office of Pediatric Therapeutics, Office of the Commissioner, US Food and Drug Administration, 10903 New Hampshire Ave, 20993 Silver Spring, MD USA; 3 Office of Therapeutic Products, Center for Biologics Evaluation and Research, US Food and Drug Administration, 10903 New Hampshire Ave, 20993 Silver Spring, MD USA

**Correction to: Orphanet Journal of Rare Diseases (2024) 19:86**

10.1186/s13023-024-03097-x

Following publication of the original article [1], we have been notified that there should be a disclaimer published in the Acknowledgement note.

**Acknowledgement note now is**:

## Acknowledgements

The authors would like to thank Sandra Retzky and Dionna Green for their input on this research project.

**Acknowledgement note should be**:

## Acknowledgements

The authors would like to thank Sandra Retzky and Dionna Green for their input on this research project.

This manuscript reflects the views of the authors and should not be construed to represent FDA’s and the Department of Health and Human Services (HHS)’s views or policies.
